# A Scene Knowledge Integrating Network for Transmission Line Multi-Fitting Detection

**DOI:** 10.3390/s24248207

**Published:** 2024-12-23

**Authors:** Xinhang Chen, Xinsheng Xu, Jing Xu, Wenjie Zheng, Qianming Wang

**Affiliations:** 1 College of Quality & Standardization, China Jiliang University, Hangzhou 310018, China; cxh1165894497@163.com; 2 State Grid Huzhou Electric Power Supply Company, Huzhou 313000, China; xujingnow@163.com; 3 Automation Department, North China Electric Power University, Baoding 071003, China; zhengwj0726@163.com (W.Z.); qianmingwang@ncepu.edu.cn (Q.W.)

**Keywords:** deep learning, object detection, transmission line fittings, scene knowledge, context information

## Abstract

Aiming at the severe occlusion problem and the tiny-scale object problem in the multi-fitting detection task, the Scene Knowledge Integrating Network (SKIN), including the scene filter module (SFM) and scene structure information module (SSIM) is proposed. Firstly, the particularity of the scene in the multi-fitting detection task is analyzed. Hence, the aggregation of the fittings is defined as the scene according to the professional knowledge of the power field and the habit of the operators in identifying the fittings. So, the scene knowledge will include global context information, fitting fine-grained visual information and scene structure information. Then, a scene filter module is designed to learn the global context information and fitting fine-grained visual information, and a scene structure module is designed to learn the scene structure information. Finally, the scene semantic features are used as the carrier to integrate three categories of information into the relative scene features, which can assist in the recognition of the occluded fittings and the tiny-scale fittings after feature mining and feature integration. The experiments show that the proposed network can effectively improve the performance of the multi-fitting detection task compared with the Faster R-CNN and other state-of-the-art models. In particular, the detection performances of the occluded and tiny-scale fittings are significantly improved.

## 1. Introduction

The stable operation of transmission lines has a vital impact on the power system [1]. The fittings are the key components that can maintain the stability of transmission lines and are divided into protective fittings, connecting fittings, strain clamps and suspension clamps, etc., [2]. However, the fittings are susceptible to the interference of wind, rain and snow because transmission lines are usually distributed in harsh environments such as snowfields, plains and deserts. Therefore, the fittings are liable to defects such as corrosion, incline and damage [3,4,5]. So, it is necessary to inspect the fittings regularly to reduce the defects for the stable operation of transmission lines.

With the widespread application of unmanned aerial vehicle (UAV) inspection technology in transmission lines [6,7,8], the workload of operators has been significantly reduced. However, the large volume of aerial images captured by UAVs still requires manual inspection, which limits the overall efficiency Hence, it is essential to introduce intelligent detection technologies with transmission line components. The fittings, as one of the fundamental components of transmission lines [9], are responsible for greatly improving the efficiency of power maintenance [10], reducing the burden of operators and realizing the defect detection of the fittings.

**Figure 1:**
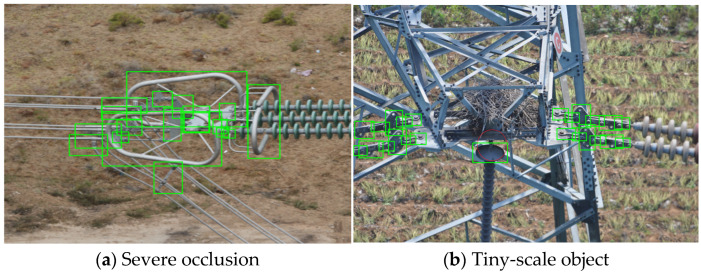
Fitting object detection problems (Objects are indicated by green boxes).

At present, most state-of-the-art object detection methods [11,12,13] view partial regions of images superficially without using the domain knowledge of the object. These methods require high-quality feature representations of region proposals to achieve satisfactory detection results. However, in the multi-fitting detection task, the feature quality of region proposals is influenced by the following two issues:

(1) Severe occlusion. Generally speaking, there are varying degrees of occlusion between the fittings due to the cameras’ shooting angles and the fittings’ connection modes. As shown in Figure 1a, the fittings such as yoke plates, u-type hanging rings and hanging boards are occluded by the shielded rings, which leads to a lack of the occluded region features. At the same time, the region proposal of shielding rings has noise features due to the existence of other fittings.
(2) Tiny-scale object. As shown in Figure 1b, the tiny-scale fittings such as hanging boards and u-type hanging rings account for a miniature proportion in the whole image due to the influence of the camera range and the scale of the fittings, resulting in less information on the region proposal features.

Both of the problems above will cause a decline in the quality of the region proposal features. Accordingly, multi-fitting detection still has great potential for making further progress.

**Figure 2:**
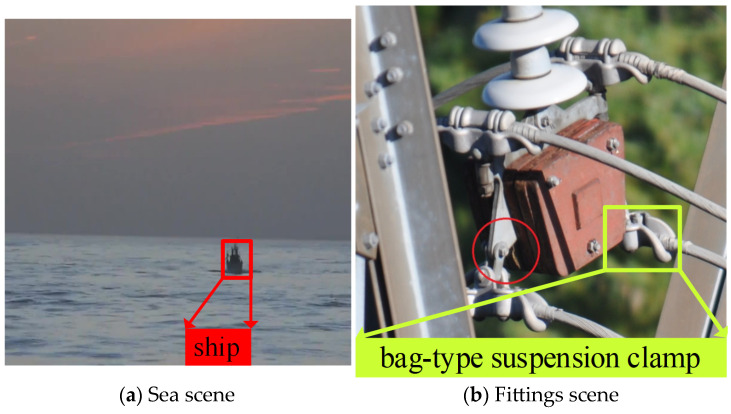
Comparison chart of scene meanings.

Most visual concepts in natural images consist of scenes, objects and relationships [14]. Psychology indicates that scene knowledge and object relationships play a vital role in the process of object recognition by humans. General scene knowledge has been validated to adequately improve the effect of object detection in the literature [14,15,16,17,18,19]. As shown in Figure 2a, if humans cannot directly identify the ship (object) in the image, they will tend to utilize the sea (scene) to infer the object. Obviously, the object at sea is more likely to be a ship than other transportation methods such as cars, trains, etc. In traditional object detection tasks, general scene knowledge often plays a vital role in improving detection accuracy by leveraging the relationships between objects and their surroundings. However, in the context of multi-fitting detection for transmission lines, the definition and application of scene knowledge present unique challenges. Unlike natural scenes, where the background (e.g., sea, forest) can provide strong contextual clues, the transmission line inspection scene lacks such explicit correlations. Here, the “scene” is redefined as the aggregation of fittings, emphasizing the domain-specific relationships and structural arrangements among fittings. Expert knowledge from the power industry, such as the typical combinations and spatial arrangements of fittings, can be integrated.

Hence, we define the scene as the aggregation of the fittings in the multi-fitting detection task. As shown in Figure 2b, the scene in the image is an anti-dancing scene. Experienced operators can infer the occluded fittings in the green box according to the visual features and structural characteristics. Therefore, in a multi-fitting detection task, the scene defined by this method will lead to scene knowledge, including global context information, fitting fine-grained visual information and structure information between fittings. As a result, unlike previous detection tasks [11,12,13], our method mainly considers the severe occlusion problem and tiny-scale object problem and utilizes the scene knowledge containing domain information to improve the effect of multi-fitting detection. Specifically, the proposed SKIN incorporates a scene filter module (SFM) to extract global context and fine-grained fitting information by filtering noise and highlighting relevant features. Additionally, the scene structure information module (SSIM) models the structural relationships among fittings using a scene-fitting prior matrix derived from professional knowledge. These innovations ensure that occluded and tiny fittings, often missed by general-purpose object detection methods, are effectively identified by leveraging scene-level domain knowledge. Compared with existing approaches that treat fitting detection as a generic object detection problem, the proposed method significantly enhances detection performance by embedding task-specific knowledge into the model architecture.

**Figure 3:**
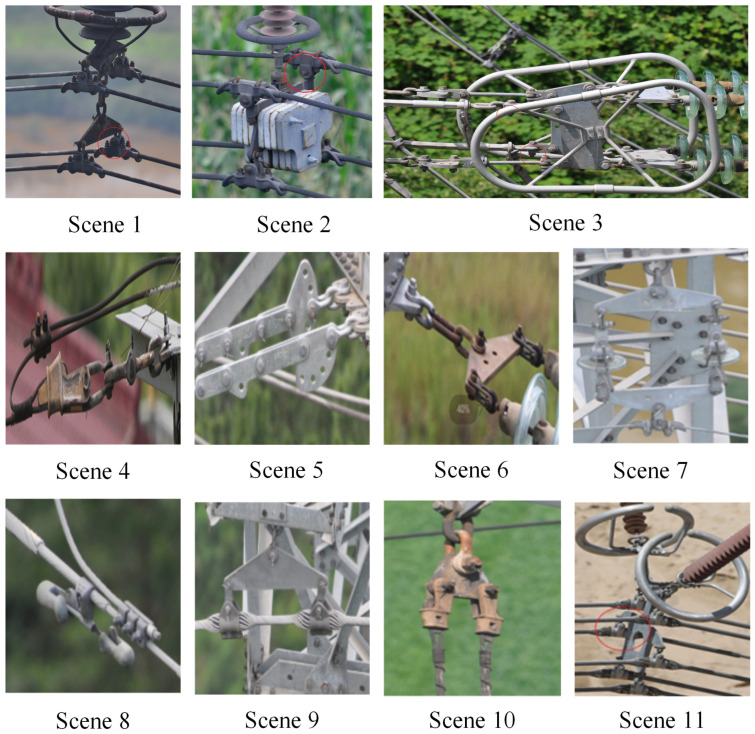
Scene definition diagram.

The main contributions of our papers can be summarized as follows:

(1) We define the aggregation of fittings as a “scene,” incorporating electrical power industry knowledge and operators’ recognition habits to improve multi-fitting detection. Hence, as shown in Figure 3, we exemplify eleven common scenes to assist the multi-fitting detection. The Scene Knowledge Integrating Network (SKIN) integrates this knowledge through the scene filter and scene structure information modules.
(2) The scene filter module collects global context and fine-grained visual information using a Gated Recurrent Unit (GRU), encoding it into scene features and passing it to the scene semantic features for further processing.
(3) The scene structure information module encodes and learns scene structure information from a scene-fitting prior matrix, integrating this information with the scene semantic features to improve the detection of occluded and small fittings.

## 2. Related Work

### 2.1. Fitting Detection

The classical methods of image processing and machine learning were used in the early stage of the research on fitting detection technology. Some of these classical methods identified the fittings directly based on the morphology [20,21,22,23] of different categories of the fittings, and some of them used classical machine learning algorithms for recognition by extracting artificial features. For example, Liu et al. [24] applied the cascade classifier and the support vector machine (SVM) to complete fitting detection by extracting the Harr-Like features and the HOG features from images. Guo et al. [25] designed the multi-cycle and multi-class method based on SVM for fitting detection. However, the reliance on handcrafted features and basic models in these classical methods resulted in weak feature extraction capabilities and limited resistance to interference, leading to unsatisfactory fitting detection performance.

With the development of deep learning, more and more attention has been paid to the research of fitting detection based on deep learning. For example, in order to improve the detection effect of shockproof hammers and grading rings, Tang et al. [26] expanded the dataset by image rotation and adjusted the kernel size of the convolution neural network. Zhang et al. [27] proposed an improved training method based on transfer learning in the yolov3 model to reduce the dataset requirement of the model. As a result, the few-shot problem about clamps and shockproof hammers was solved and the accuracy and generalization of fitting detection were improved. Jiao et al. [28] enhanced the shockproof hammer dataset by the joint expansion of samples and label files to improve the recognition rate. Nevertheless, the above studies were mainly aimed at a few categories of fittings and focused on data augmentation. They did not integrate the overall rules of transmission lines and the characteristics of the fittings into the detection tasks.

### 2.2. Object Detection Model Integrating Knowledge

In recent years, some approaches based on deep learning have attempted to apply object–object or scene–object relationships in object detection tasks. For instance, in order to improve the detection network, Zheng et al. [18] modeled the object proposal features and the scene features as nodes and modeled the prior knowledge of object–object relationship and object-scene relationship as edges. Hang et al. [29] introduced global semantic pools by utilizing the weight of a classifier, and the high-level semantic representations of different categories in the global semantic pool were updated through attribute and relation. Then, the adaptive global reasoning module was used to obtain the enhanced features to assist the classification and regression. Chen et al. [30] introduced the concepts of image-level context and instance-level context and then modeled the relationship between different objects by proposing a spatial memory network (SMN). Liu et al. [14] proposed a structure inference net (SIN), in which the object–object relationship and scene knowledge were utilized by a GRU. Jiang et al. [31] proposed hybrid knowledge routed modules (HKRM) to integrate explicit knowledge and implicit knowledge into the model by using an explicit knowledge module and an implicit knowledge module. The networks mentioned above mainly modeled the relationships between objects to introduce knowledge, and a part of them included scene knowledge to assist detection. However, the common scene knowledge that cannot be applied to multi-fitting detection was utilized above.

## 3. Methods

### 3.1. Overview

**Figure 4:**
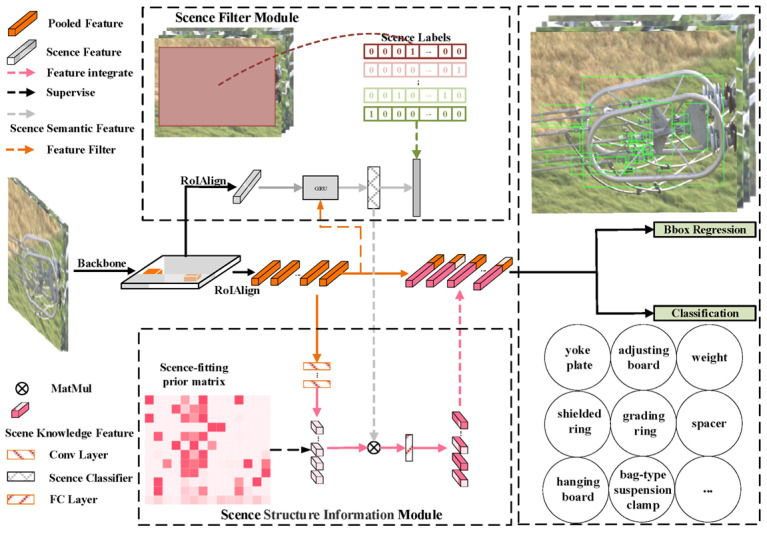
SKIN model structure.

The overall network architecture of this paper is shown in Figure 4. Firstly, the processed aerial image of the fittings is utilized as the input of the network. Then, the convolutional neural network resnet101 [32] is utilized as the backbone for feature extraction, and the original scene feature and the region proposal features are obtained by using the region proposal network (RPN) and the pooling layer. The original scene feature learns the global context information and fitting fine-grained visual information by the scene filter module in the supervised learning method and transmits the learned information to the scene semantic features. The scene structure information module supervises the region proposal features, learning the scene structure information from the scene-fitting prior matrix, and promotes the combination of the scene semantic features and the learning results to obtain the relative scene features of region proposals. Finally, these relative scene features are used for feature mining, and the results are integrated into the region proposal features. As a result, the scene knowledge from the relative scene features assists the network in inferring occluded and tiny-scale objects, thereby enhancing multi-fitting detection.

### 3.2. Scene Filter Module

#### 3.2.1. The Generation of the Original Scene Feature

The scene filter module requires the original scene feature as input, so it is necessary to generate the original scene feature in the first place. Our model is based on Faster R-CNN, whose former part can be summarized as follows:

(1) $$F_{CNN}=\Gamma_{CNN}(Z_{i}||\theta_{CNN})$$

(2) $$K_{rois}=\Gamma_{RPN}(F_{CNN}||\theta_{RPN})$$

where $Z_{i}$ is the i-th input image, $\Gamma_{CNN}(\cdot ||\theta_{CNN})$ is the process of feature extraction and $\theta_{CNN}$ is the learnable weight in this process. The base features $F_{CNN}$ are utilized to obtain region proposals $K_{rois}$ by region proposal network $\Gamma_{RPN}(\cdot ||\theta_{RPN})$, in which $\theta_{RPN}$ is the learnable weight.

Inspired by [14], we introduce the global region for generating the original scene feature. After that, the current region proposals can be represented as

(3) $${K^{\prime }}_{rois}={\left\{(x_{0}^{j},y_{0}^{j},x_{1}^{j},y_{1}^{j})\right\}}_{j=1}^{N_{\eta }+1}$$

where $\left(x_{0}^{j},y_{0}^{j}\right)$ means top-left coordinate of the j-th region and $\left(x_{1}^{j},y_{1}^{j}\right)$ means bottom-right coordinate of the j-th region. $N_{\eta }$ is the number of the region proposals and the extra one is the global region.

Then, the current region proposals are utilized to realize pooling operation on the base features, and the results are input into the fully connected layer to obtain the region proposal features and the original scene feature. This process can be expressed as

(4) $$F_{tail}=\Gamma_{tail}([F_{CNN},{K^{\prime }}_{rois}]||\theta_{tail})\in R^{(N_{\eta }+1)\times C_{\gamma }}$$

where $\Gamma_{tail}(\cdot ||\theta_{tail})$ represents the pooling layer and the fully connected layer and $\theta_{tail}$ is the learnable weights. After that, $F_{tail}$ can be sliced into the region proposal features $F_{prop}\in R^{N_{\eta }\times C_{\gamma }}$ and the original scene feature $F_{sc}\in R^{1\times C_{\gamma }}$. In addition, $C_{\gamma }$ is the dimension of the features.

#### 3.2.2. The Filtering of the Original Scene Feature

Although the original scene feature contains the global context information and the fitting fine-grained visual information, there is a lot of information noise due to the small proportion of effective fitting objects and large proportion of invalid background regions in UAV aerial images.

Thus, the scene filtering module is designed to filter the original scene features, removing redundant noise and emphasizing the fine-grained visual details of the fittings and the relevant global context information. The information used to filter the original scene features can be expressed as follows:

(5) $$F_{prop}={\left\{f_{prop}^{m}\right\}}_{m=1}^{N\eta }\to F_{fore}={\left\{f_{prop}^{n}\right\}}_{n=1}^{N_{\mu }}$$

where → represents the selecting process and $N_{\mu }$ is the number of region proposals in the selecting process. It means that the features $F_{fore}$, which contain the region proposal features of the fittings as possible, are selected from $F_{prop}$.$f_{prop}^{m}$ is the m-th feature in the $F_{prop}$ and $f_{prop}^{n}$ is the n-th feature in the $F_{fore}$.

From an individual perspective, a single region proposal feature represents the fine-grained visual information of a specific fitting within the image scene. In contrast, from a global perspective, the global context information is derived from the collective set of multiple region proposal features across the entire scene.

To effectively capture both the fine-grained visual details and the global context, a memory unit is needed. This memory unit sequentially processes each region proposal feature one by one, allowing it to retain the relevant fine-grained visual information of each fitting encountered. By sequentially aggregating these features, the memory unit can ultimately capture the global context composed of multiple region proposals within the scene.

However, in the process of aggregating these features, some background features may be present. These background features often contain irrelevant or invalid information that can introduce noise and reduce detection accuracy. Therefore, the memory unit must also have the capability to filter out or forget invalid background information while retaining the important fitting features.

To achieve this, we employ a mechanism that enables the memory unit to selectively remember useful information and discard irrelevant features, ensuring that the aggregated global context information remains accurate and meaningful for the multi-fitting detection task.

**Figure 5:**
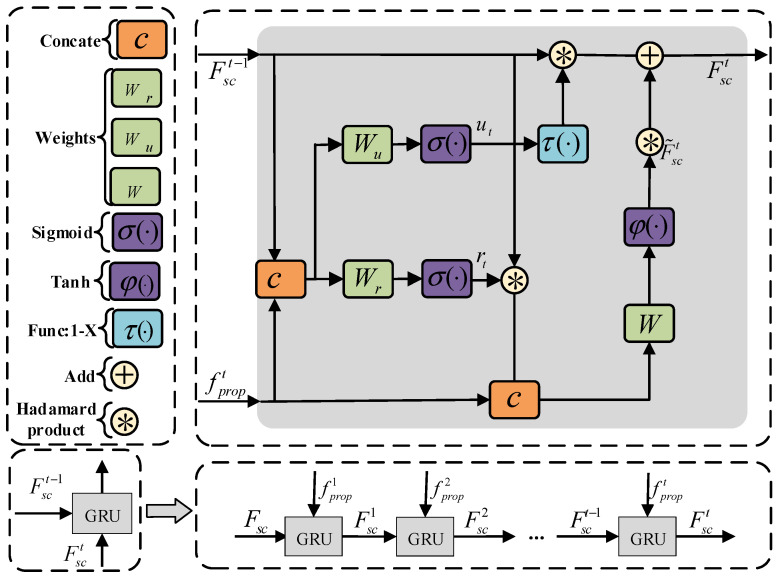
Scene filtering module structure diagram.

So, we select a Gated Recurrent Unit (GRU) [33], which has a simple structure, is easy to combine and has excellent long-term memory. Then, as shown in Figure 5, the original scene feature is filtered by utilizing GRU:

Firstly, the input is constructed as a sequence $F_{fore}={\left\{f_{prop}^{t}\right\}}_{t=1}^{N\mu }$, in which t represents the t-th moment. $f_{prop}^{t}$ is the single feature inputted at the t-th moment, and the original scene feature $F_{sc}$ is taken as the initial state of GRU. Then, the update gate $u_{t}$ at the t-th moment can be represented as

(6) $$u_{t}=\sigma (W_{u}\cdot [F_{sc}^{t-1},f_{prop}^{t}])$$

where σ(~) is the sigmoid activation function, $F_{sc}^{t-1}$ is the state of the t−1 moment, [~,~] is the concatenation of matrices and $W_{u}$ is the learnable weights. Accordingly, using the update gate $u_{t}$, the scene state $F_{sc}^{t}$ at the t-th moment can be obtained:

(7) $$F_{sc}^{t}=(1-u_{t})*F_{sc}^{t-1}+u_{t}*{\tilde{F}}_{sc}^{t}$$

where ∗ represents the Hadamard product. Further, the formula of ${\tilde{F}}_{sc}^{t}$ is

(8) $${\tilde{F}}_{sc}^{t}=\varphi (W\cdot [r_{t}*F_{sc}^{t-1},f_{prop}^{t}])$$

where φ(~) is the tanh activation function, $r_{t}$ is the reset gate at the t-th moment and W is the learnable weights. The reset gate determines how the new input information is combined with the previous memory, and its formula is as follows:

(9) $$r_{t}=\sigma (W_{r}\cdot [F_{sc}^{t-1},f_{prop}^{t}])$$

where $W_{r}$ is the learnable weights.

After the above process, the filtered scene feature ${F^{\prime }}_{sc}=F_{sc}^{t}(t=N_{\mu })$ can be obtained through the collaborative control of the update gate and reset gate.

#### 3.2.3. The Constraint of the Scene Filtering

The scene filter module is designed in a supervised learning method to constrain the GRU’s memory direction (emphasizing the effective global context information and fitting fine-grained visual information and ignoring invalid background information). The process is as follows:

(1) First, the scene label space is constructed. Specifically, the scene label $Y_{i}=\left\{y_{i}^{0},y_{i}^{1},\cdots ,y_{i}^{n_{s}}\right\}\in R^{n_{s}+1}$ is assigned to the i-th image $Z_{i}$. In addition, $n_{s}$ is the number of scene categories and the extra dimension represents the situation of no scene. When the image contains the scenes, the values of their corresponding dimension are 1, and otherwise they are 0. And if the image does not contain the scenes, the $y_{i}^{0}$ is 1.
(2) Second, the scene classifier is constructed for completing the scene classification task. The filtered scene feature is mapped into the scene label space through the scene classifier:
  (10) $${\hat{Y}}_{i}=\sigma ({F^{\prime }}_{sc}\cdot W_{ca})=\left\{{\hat{y}}_{i}^{0},{\hat{y}}_{i}^{1},\cdots ,{\hat{y}}_{i}^{n_{s}}\right\}\in R^{n_{s}+1}$$
  where $W_{ca}\in R^{C_{\gamma }\times (n_{s}+1)}$ is the weight of the scene classifier. And the distribution of ${\hat{Y}}_{i}$ in the scene label space is urged to approach $Y_{i}$ by the following loss function:
  (11) $$L_{sc}=-\frac{1}{N_{b}}\frac{1}{N_{s}}\sum_{m=1}^{N_{b}}\sum_{n=1}^{N_{s}}(y_{m}^{n}\log({\hat{y}}_{m}^{n})+(1-y_{m}^{n})\log(1-{\hat{y}}_{m}^{n}))$$
  where $N_{s}$ means $n_{s}+1$, and $N_{b}$ is the number of images contained in a batch.

Hence, in order to complete the scene classification task, GRU will be constrained to remember the valid information and ignore the invalid information in the process of backpropagation. And the scene classifier weights will obtain the information during the mapping process. Therefore, referring to [29], we extract the scene classifier weight to form the scene semantic feature pool, which is similar to the memory when humans recall a category. So, the information can be transmitted into the semantic concepts of all scenes (not just the scenes that appear in the image) in the memory. Further, the scene filtering process is equivalent to the human viewing each image, and the semantic concept of the scene categories in memory is modified based on the difference in the image. Accordingly, the scene semantic features are also compatible with the specificity of each image.

### 3.3. Scene Structure Information Module

#### 3.3.1. Scene-Fitting Prior Matrix Construction

The main function of the scene structure information module is to learn the structure information between the fittings and combine the information with the scene semantic features to generate the relative scene features. The structure information can be extracted in different ways: from the perspective of instance-level, it is the structure relation between the instance-level fittings and can be represented as the fitting-fitting co-occurrence matrix [34,35] or spatial location matrix [36]; from the perspective of scene-level, the structure information is the combination of the fittings included in the scene. Therefore, we construct the scene-fitting prior matrix using conditional probability to represent the scene-level structural information.

Hence, the conditional probability formula for constructing the scene-fitting prior matrix is

(12) $$P(fit_{i}|sc_{j})=\frac{P(fit_{i},sc_{j})}{P(sc_{j})}$$

This formula represents the probability of the i-th fitting when the j-th scene appears. There are $n_{s}+1$ categories of scenes, and no scene (consisting of scattered fittings that cannot be classified as any of the scenes in the definition) is also a category of scenes. The elements of the above formulate can be represented as

(13) $$P(fit_{i},sc_{j})=\frac{N_{ij}}{N_{all}}$$

(14) $$P(sc_{j})=\frac{N_{j}}{N_{all}}$$

where $N_{ij}$ is the number of the j-th scene which contains the i-th fitting, $N_{j}$ is the number of the j-th scene and $N_{all}$ is the number of all scenes. Further, the conditional probability formula for constructing the scene-fitting prior matrix can be represented as

(15) $$P(fit_{i}|sc_{j})=\frac{N_{ij}}{N_{j}}$$

**Figure 6:**
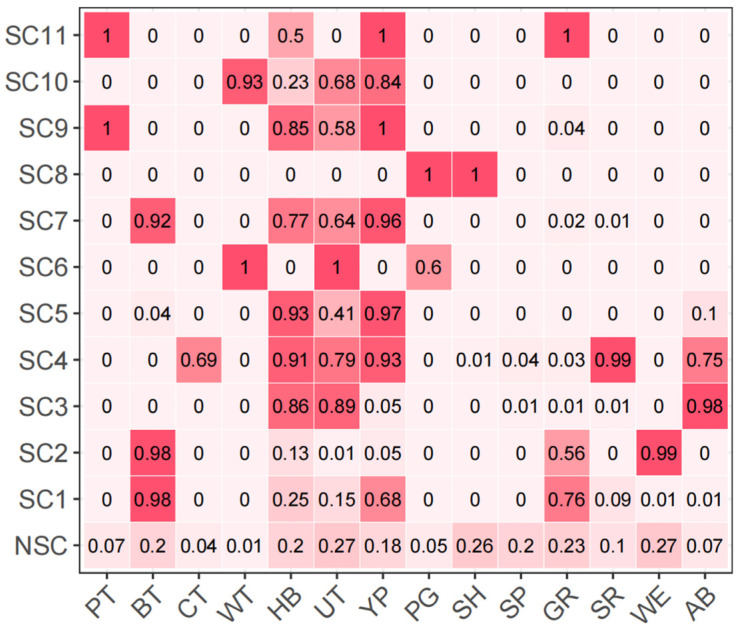
Scene-fitting co-existence matrix.

Therefore, the scene-fitting prior matrix $M_{s-f}\in R^{(n_{s}+1)\times n_{f}}$ can be constructed after counting the $N_{ij}$ and $N_{j}$. As shown in Figure 6, all eleven categories of scenes have obvious rules for the fitting combination (scene-level structure information), except no scene.

#### 3.3.2. The Network Structure of SSIM

**Figure 7:**
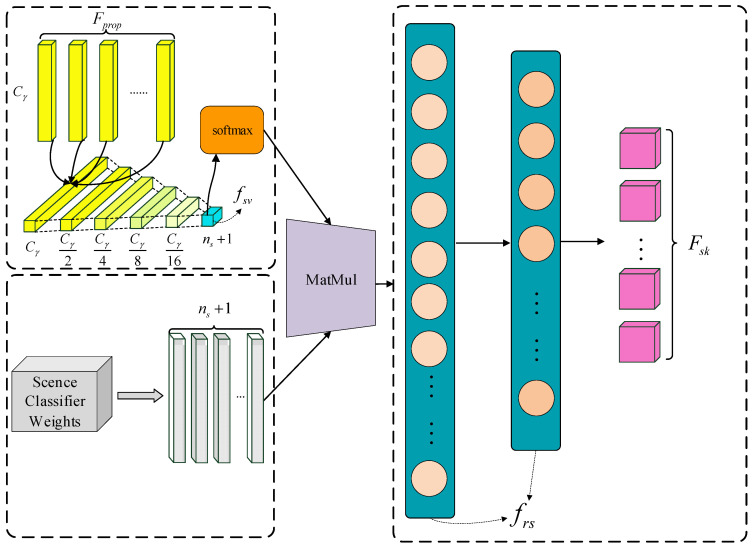
The network structure of SSIM.

As shown in Figure 7, referring to [31], we propose the scene structure information module to learn the scene-level structure information. However, the scene-level structure information learned in this process depends on the statistical results and lacks compatibility with each image. Therefore, the specific information contained in the scene semantic features from the scene filtering module should be transmitted to the scene structure information module. This allows for the extraction of the relative scene features of the regions. Consequently, the relative scene features will encompass global context information, fine-grained visual details of the fittings and scene structure information, in line with the definition of scene knowledge presented in this paper. Then, the relative scene features are used for feature mining, and the results are integrated into the region proposal features for improving fitting detection. The process of the scene structure information module is as follows:

(1) Obtain the ground-truth scene vectors. There are the ground-truth categories $GT_{cls}\in R^{N_{\eta }\times 1}$ corresponding to the region proposal features $F_{prop}\in R^{N_{\eta }\times C_{\gamma }}$. Hence, the ground-truth scene vectors $GT_{\mathrm{sv}}={\left\{gt_{sv}^{n}\right\}}_{n=1}^{N_{\eta }}\in R^{N_{\eta }\times (n_{s}+1)}$ can be obtained through the map of $GT_{cls}$ on $M_{s-f}$ and $gt_{sv}^{n}$ is one of the ground-truth scene vectors. This explicit mapping using $GT_{cls}$ will ensure the information extraction and provide a stable guarantee for the supervised learning of the scene-level structure information.

(2) Learn the scene-level structure information. The information is learned by constructing the multiple convolution layers:

(16) $$F_{sv}=Convs_{sv}(F_{prop})$$

where $F_{sv}$ is the predicted scene vectors and can be represented as ${\left\{f_{sv}^{n}\right\}}_{n=1}^{N_{\eta }}\in R^{N_{\eta }\times (n_{s}+1)}$. $f_{sv}^{n}$ is one of the predicted scene vectors, and $Convs_{sv}(\sim )$ means the multiple convolution layers. Further, the scene knowledge module will learn the scene-level structure information contained in the scene-fitting matrix by completing the task of urging $F_{sv}$ to approach $GT_{\mathrm{sv}}$. The loss function in this task is as follows:

(17) $$L_{sv}(F_{sv},GT_{sv})=\frac{1}{N_{b}}\sum_{n=1}^{N_{b}}\sum_{m=1}^{N\eta }{\left\|\left.{\left(f_{sv}^{m}\right)}_{n}-{\left(gt_{sv}^{m}\right)}_{n}\right\|\right.}_{1}$$

where ${\left\|\left.∼\right\|\right.}_{1}$ is the 1-norm of vector.

(3) Obtain the relative scene features. The scene structure information learned in the previous step depends on the statistical results, while ignoring the specific visual features of the fittings and the specific combination characteristics in each image (fitting fine-grained visual information and global context information). Therefore, it is necessary to transmit the specific information of the scene semantic features from the scene filter module to the results of the scene structure information module:

(18) $$F_{rs}=\psi (F_{sv})\cdot {\left(W_{ca}\right)}^{T}$$

where ψ(~) is the softmax activation function, ${\left(\sim \right)}^{T}$ is the transpose of matrix, $F_{rs}\in R^{N_{\eta }\times C_{\gamma }}$ is the relative scene features and $W_{ca}$ is the learnable parameters. Because the multiple fittings aggregate into a scene, the fitting will have the relative scene feature. The fitting’s relative scene feature is affected by the fitting fine-grained visual information of other fittings in the image, the global context information of the fitting’s location in the image and the structure information between the fitting and other fittings in the image, conforming to the definition of scene knowledge. The above three categories of information will affect each other in different images in order to make the relative scene features specific.

(4) Feature mining and knowledge integration. Finally, $F_{rs}$ is utilized for feature mining to obtain the scene knowledge features $F_{sk}$:

(19) $$F_{sk}=F_{rs}\cdot W_{sk}$$

where $W_{sk}\in R^{C_{\gamma }\times C_{e}}$ is the weight of the transformation matrix. So, $C_{e}$ is the dimension of the scene knowledge features. The combined features obtained by combining $F_{prop}$ and $F_{sk}$ in cascade way are input into the classification detector and the regression detector to assist in the inference of the occluded fittings and the tiny-scale fittings.

## 4. Experiment

### 4.1. Experiment Settings

#### 4.1.1. Dataset Description

The multi-fitting dataset, which is taken by UAV from the inspection site, is selected. It contains fourteen categories of fittings, including pre-twisted suspension clamp (PT), bag-type suspension clamp (BT), compression-type strain clamp (CT), wedge-type strain clamp (WT), hanging board (HB), u-type hanging ring (UT), yoke plate (YP), parallel groove clamp (PG), shockproof hammer (SH), spacer (SP), grading ring (GR), shielded ring (SR), weight (WE) and adjusting board (AB). As shown in Table 1, The train set has 1330 images, containing 16,358 fitting objects. The test set has 318 images, containing 2767 fitting objects.

#### 4.1.2. Experiment Environment and Hyperparameter Setting

The Scene Knowledge Integrating Network is trained and tested on an NVIDIA RTX3090 professional accelerator card with 24 GB of VRAM, which is headquartered in Santa Clara, California, USA., ensuring sufficient computational power for large-scale deep learning experiments. The operating system is Ubuntu 18.04.5 LTS, with CUDA 11.2 used to optimize GPU-based training. The implementation is written in Python 3.8, utilizing the PyTorch framework (version 1.x), which provides a flexible and efficient platform for building and training deep learning models.

To verify the effectiveness of the proposed Scene Knowledge Integrating Network (SKIN), Faster R-CNN with ResNet101, pretrained on the ImageNet dataset, is used as the baseline model. ResNet101 serves as the backbone network for feature extraction, leveraging its deep architecture to extract high-quality features. The hyperparameters for the region proposal network (RPN) are set to 128 during training and 300 during testing, ensuring sufficient candidate proposals for accurate detection.

The dataset is augmented using image inversion, which enhances diversity in training samples and improves model robustness. The training process employs the stochastic gradient descent (SGD) algorithm with a momentum of 0.9 to stabilize convergence. The initial learning rate is set to 0.0034, which decays by 10% every 15 epochs to allow fine-tuning as training progresses. The total number of epochs is 20, balancing between convergence and computational cost.

The scene filter module (SFM) selects 40 region proposal features for filtering, which are processed to extract global context and fine-grained fitting visual information. In the scene structure information module (SSIM), the dimension of the scene knowledge features is set to 256, providing sufficient capacity to represent structural relationships and domain-specific scene knowledge effectively.

The experiments are conducted under controlled conditions to ensure reproducibility, with random seeds set for initialization and data splitting. Performance metrics, including mAP^50^, AP^50^, and AR, are used to evaluate the effectiveness of the proposed method in comparison to the baseline and other models. Our code is available at the following link: https://github.com/CharmingWang/SKIN (accessed on 15 December 2024).

### 4.2. Comparison with State-of-the-Art Models

**Figure 8:**
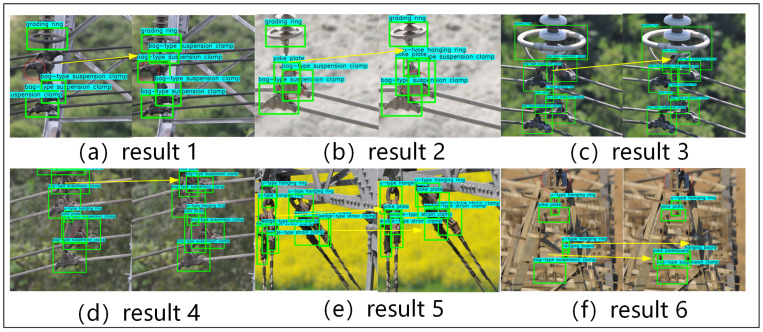
Qualitative result comparison on fitting dataset.

To verify the improvements in model performance brought by the proposed scene filter module and scene structure information module, as well as the advantages over current general object detection algorithms, this paper compares the proposed method with commonly used object detection methods at present. The comparison methods include SSD [37], RetinaNet [38], YOLOv5 [39], YOLOv8 [40], R-FCN [41], EfficientNetv2 [42] and MobileNetv2 [43], as well as some object detection models based on the Transformer architecture [44], such as Swin Transformer [45], DETR [46], DINO [47] and CO-DETR [48]. The results are shown in Table 2. The model that incorporates the SFM and SSIM modules has higher AP50 values in categories such as BT, WT and SH compared to current object detection algorithms like YOLOv8, DETR and other mainstream methods. Additionally, the detection accuracy for categories like PT and SR is also close to the optimal value. Figure 8 presents a visual comparison between the proposed algorithm and other algorithms.

The experimental results show that our algorithm performs better than other general-purpose object detection algorithms on multiple indicators, especially with significant performance improvement in complex scenarios. Compared with the baseline model, the improved model has improved detection accuracy in most categories, and has achieved a 4.9% improvement in overall detection accuracy. This is because the introduction of the scene filtering module and the scene knowledge module enables the algorithm to have a deeper understanding of the special scene of transmission line fittings, thereby improving the detection performance.

Compared with traditional models, such as SSD and RetinaNet, and Transformer-based models, such as Swin Transformer and DETR, the method proposed in this paper has achieved significant advantages in the detection of most categories. Compared with advanced YOLOv8, Swin Transformer and DETR, mAP50 has increased by 0.9%, 1.1% and 3.7%, respectively. This shows that the scene filtering module effectively filters out large proportions of invalid areas, reduces noise interference and improves overall detection accuracy. Moreover, the scene knowledge module enhances the generalization ability of the model for different scenes and environments by learning the scene-fittings co-occurrence matrix and prior knowledge. The experimental results for testing speed are shown in Table 2. The proposed model cannot achieve the best performance. However, In current power line inspection workflows, unmanned aerial vehicles (UAVs) are typically used to capture large volumes of image data, which are then transmitted to local servers for processing. This approach leverages local computing resources to perform detailed and accurate analysis, ensuring that the detection results meet the stringent precision requirements of real-world applications. Although our method may not achieve the fastest detection speed, it satisfies the processing speed requirements for local server-based analysis and provides the high-accuracy results necessary for practical deployment in transmission line inspection tasks.

As shown in Figure 8, in each subfigure, the left side displays the test results of the baseline model, while the right side shows the test results of the proposed model. The yellow line indicates the change in the comparison of test results. From the figure, it can be seen that our proposed model significantly improves the missed detections and false detections of dense targets. The specific improvements are as follows:

(1) In subfigure (a), our model detects two inverted bag-type suspension clamps.
(2) In subfigure (b), our model detects small fitting targets connecting the grading ring and the yoke plate, specifically the u-type hanging ring.
(3) In subfigures (c) and (d), our model detects the occluded link plates.
(4) In subfigure (e), our model detects the previously missed yoke plate, and the misdetected wedge-type strain clamp bounding box is also correctly rectified.
(5) In subfigure (f), our model corrects the false detection of a u-type hanging ring and accurately detects the previously missed yoke plate.

These improved detection results are attributed to our model’s ability to utilize scene information within the image to infer which fittings are more likely to appear, thereby producing more accurate results.

### 4.3. Ablation Analysis

Taking the Faster R-CNN as the baseline model, we conducted detailed experiments to prove the effectiveness of different modules and different hyperparameters in different modules in the multi-fitting detection task.

(1) Verify the effectiveness of the module. Based on Faster R-CNN, we added a scene filter module and scene structure information module in turn in order to verify the effectiveness of scene filter module and scene structure information module. As shown in Table 3, the AP^50^ value of adding SFM increases by 3.3% compared with the baseline mode. And the SSIM mainly supplements the scene structure information that the SFM has not learned, so it cannot be used alone. Therefore, the effectiveness of the SSIM is reflected by adding the SSIM on the basis of the SFM. So, as shown in Table 3, the AP^50^ value of the network with the SFM and the SSIM is increased by 4.8%, which is 1.5% higher than that of the network with the SFM. Further, the experiments above also explain the effectiveness of the global context information and fitting fine-grained visual information mined by the SFM and the effectiveness of the scene structure information mined by the SSIM.

(2) The effect of different prior matrices. In order to verify the validity of the scene-fitting prior matrix, we design the ones prior matrix and random prior matrix used in SSIM. As shown in Table 4, the effects of using the ones prior matrix and random prior matrix are obviously lower than the effect of using scene-fitting prior matrix and also lower than the effect of using SKIN only with SFM. Those show that the wrong prior matrix will mislead the learning direction of SKIN, make the SKIN learn the wrong scene structure information and lead to the poor effect.

(3) The effect of different $N_{\mu }$ values in the SFM. In the scene filter module, we utilize GRU to filter the original scene feature. The $N_{\mu }$ region proposal features are sequenced, and retained to learn fitting fine-grained visual information and global context information in the filtering process. As shown in Table 5, we tested the effect of different $N_{\mu }$ values when the dimension of the scene knowledge features is 256. It can be seen that the SKIN obtains the best effect when $N_{\mu }=40$. This is because, when the $N_{\mu }$ value is too small, the original scene feature only remembers part of the fitting fine-grained visual information and global context information in the image through GRU, and a lot of information is missed. When the $N_{\mu }$ value is too large, there will be too much invalid background information which GRU cannot completely filter out, resulting in the noise information affecting the detection effect of the SKIN.

(4) The effect of different scene knowledge feature dimensions. In the scene structure information module, the relative scene features containing scene knowledge are utilized for feature mining and feature integration, and the dimension of the feature mining is determined by $C_{e}$. As shown in Table 6, when $N_{\mu }=40$, we test the effects of different $C_{e}$ values. And the SKIN obtains the best effect when $C_{e}=256$. This is because, when the $C_{e}$ value is too small, it is not enough to cover the scene knowledge obtained by feature mining. When the $C_{e}$ value is too large, there is much redundant information and the dimension of the combined features is too high. So, it is strenuous for the classifier and the regression to extract the vital information from high-dimension features, which affects the detection effect.

### 4.4. More Discussion

**Figure 9:**
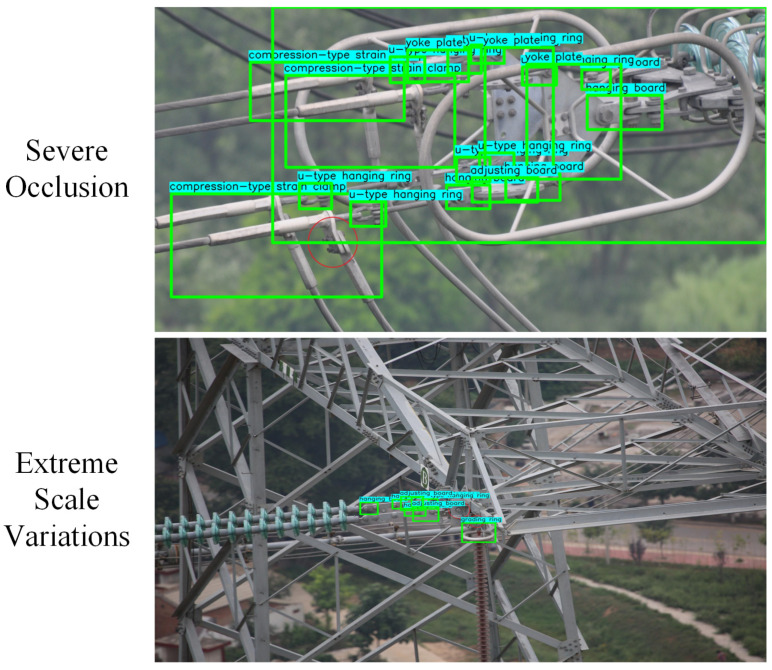
More test results.

As shown in Figure 9, we provide new examples to discuss additional limitations of the model. The figure illustrates that our model still faces challenges related to severe occlusion and extreme scale variations:

(1) Severe Occlusion: In some cases, when fittings are extensively obscured by other components, the model’s ability to infer their presence is reduced, even with the assistance of scene knowledge.
(2) Extreme Scale Variations: Very small fittings, which occupy only a few pixels, pose challenges due to limited visual information, making them harder to detect accurately.

In future work, we plan to address these issues by incorporating higher-resolution imagery or multi-scale feature fusion techniques to improve detection performance.

## 5. Conclusions

In this study, we redefined the concept of a “scene” for transmission line fittings, distinguishing it from conventional object detection scenes due to the unique structural relationships between fittings. Building on this, we proposed the Scene Knowledge Integrating Network (SKIN) to address the challenges of severe occlusion and tiny-scale objects in multi-fitting detection.

The SKIN leverages the scene filter module (SFM) to capture fine-grained visual details and global context, while the scene structure information module (SSIM) models the structural relationships among fittings. These components aggregate information through a global semantic pool, enabling more effective feature mining and integration to enhance detection performance.

The experimental results demonstrate that the SKIN achieves a 4.8% increase in mAP compared to the baseline model. Additionally, the detection performance for tiny-scale fittings improves by 11.5%, and it improves by 9.9% for severely occluded fittings. These findings highlight the effectiveness of integrating scene knowledge and provide a new approach for applying domain-specific knowledge to solve detection challenges in the power industry.

In future research, we aim to further address the challenges of severe occlusion and extreme scale variations by exploring techniques such as higher-resolution imagery, multi-scale feature fusion and 3D spatial modeling to enhance detection performance under these conditions.

## Figures and Tables

**Table 1:** Fitting dataset.

Fitting Name	Training Subset		Testing Subset		Total per Object
	#Images	#Objects	#Images	#Objects	
PT	56	98	27	50	148
BT	497	1735	150	463	2198
CT	254	923	32	110	1033
WT	24	62	12	42	104
HB	825	3800	146	577	4377
UT	707	2767	138	357	3124
YP	794	1531	161	264	1795
PG	55	64	20	24	88
SH	265	924	94	260	1184
SP	289	536	42	64	600
GR	438	701	101	153	854
SR	381	959	43	97	1056
WE	246	279	77	83	362
AB	506	1979	66	223	2202
total	1330	16,358	318	2767	19,125

**Table 2:** Comparison results with other state-of-the-art models (Bold indicates the best value in the column).

Models	mAP^50^	PT	BT	CT	WT	HB	UT	YP	PG	SH	SP	GR	SR	WE	AB	Timems/i
SSD300	51.4	78.3	85.5	40.8	11.0	29.2	23.2	58.3	5.0	82.9	69.5	92.6	71.5	97.7	54.2	8
SSD512	74.3	**91.2**	90.1	53.9	41.9	63.6	59.2	74.9	51.8	90.4	74.8	92.9	76.4	99.6	79.2	36
RetinaNet	69.8	81.2	93.2	44.9	71.3	60.0	58.3	68.9	7.7	88.1	74.0	91.4	62.9	99.3	76.6	50
YOLOv5	71.3	86.7	73.7	60.8	77.3	55.7	68.4	63.7	42.3	88.8	78.4	90.7	54.8	97.6	58.6	33
YOLOv8	75.4	85.7	77.4	69.6	74.9	60.3	74.5	71.2	**58.6**	90.7	63.9	91.6	**79.5**	98.6	59.7	127
R-FCN	67.0	76.3	35.4	59.3	73.3	57.6	48.7	78.4	52.7	72.5	62.4	87.7	69.9	94.7	68.4	230
Efficientnetv2	68.7	47.5	64.7	69.2	74.7	58.9	43.8	59.7	56.1	87.6	69.3	90.6	77.8	93.0	68.7	20
Mobilenetv2	59.4	48.5	64.5	45.3	62.8	37.7	29.6	57.8	50.3	68.7	70.8	69.4	59.6	93.5	73.6	**5**
Swin Transformer	75.2	87.4	79.4	**86.5**	73.4	69.9	**76.3**	74.2	32.9	89.8	76.3	76.1	51.2	99.8	80.2	214
DETR	72.6	74.7	73.6	62.8	67.5	**73.8**	63.6	73.7	44.2	74.3	87.5	95.2	69.7	97.6	57.7	145
DINO	75.8	91.2	87.5	58	78.2	63	72	81.5	34	80	87.5	85.5	72	90.5	79.8	210
CO-DETR	75.5	90.8	73	57.5	77.5	67.5	71	80.5	33.5	85.5	87	83.2	71.5	99	79.2	175
Baseline	71.4	81.6	89.2	56.0	64.7	49.6	49.6	78.8	33.3	81.1	86.4	89.7	62.7	**100**	76.9	158
Ours	**76.3**	91.0	**93.8**	58.6	**79.0**	48.7	52.5	**82.3**	34.8	**90.8**	**88.2**	**96.1**	72.6	**100**	**80.4**	193

**Table 3:** Ablation experiments with modules.

%	SFM	SSIM	AP^50−95^	AP^50^	AR^1^	AR^100^
Baseline			38.4	73.6	26.5	46.8
	√		41.5^+3.1^	76.9^+3.3^	27.5^+1.0^	49.6^+2.8^
Ours	√	√	42.0^+3.6^	78.4^+4.8^	27.4^+0.9^	49.9^+3.1^

**Table 4:** Results of different prior knowledge (Bold indicates the best value in the column).

Different Matrix	AP^50−95^	AP^50^	AP^75^	AR^1^	AR^100^
Ones Prior Matrix	41.1	76.4	40.4	27.3	49.7
Random Prior Matrix	41.1	76.7	40.7	27.6	49.6
Scene-Fitting Prior Matrix	**42.0**	**78.4**	**41.2**	27.4	**49.9**

**Table 5:** Results of different $N_{\mu }$ values (Bold indicates the best value in the column).

Experiments	AP^50:95^	AP^50^	AP^75^	AR^1^	AR^10^	AR^100^
$N_{\mu }=8$	41.6	76.9	41.3	27.5	49.4	49.6
$N_{\mu }=16$	41.9	77.3	41.4	27.4	49.8	49.9
$N_{\mu }=24$	41.6	77.2	41.0	27.1	49.2	49.4
$N_{\mu }=32$	41.8	77.9	40.9	27.4	49.4	49.5
$N_{\mu }=40$	**42.0**	**78.4**	**41.2**	**27.4**	**49.8**	**49.9**
$N_{\mu }=48$	41.1	77.7	41.3	27.7	49.3	49.4
$N_{\mu }=56$	41.7	77.6	42.7	27.3	49.5	49.7
$N_{\mu }=64$	42.0	77.4	41.3	28.0	49.9	50.0
$N_{\mu }=128$	41.2	77.1	40.0	27.7	49.3	49.5

**Table 6:** Results of different features $C_{e}$ (Bold indicates the best value in the column).

Experiments	AP^50:95^	AP^50^	AP^75^	AR^1^	AR^10^	AR^100^
$C_{e}=128$	40.7	76.2	39.9	27.4	49.6	49.7
$C_{e}=256$	**42.0**	**78.4**	**41.2**	**27.4**	**49.8**	**49.9**
$C_{e}=512$	41.1	77.2	41.0	27.0	49.2	49.3
$C_{e}=1024$	41.1	77.3	40.4	27.4	49.2	49.3
$C_{e}=2048$	42.0	77.0	42.5	27.9	50.1	50.2

## Data Availability

We are sorry that the power data cannot be disclosed due to its particularity and confidentiality; further inquiries can be directed to the corresponding authors.
